# MRI or ^18^F-FDG PET for Brain Age Gap Estimation: Links to Cognition, Pathology, and Alzheimer Disease Progression

**DOI:** 10.2967/jnumed.123.265931

**Published:** 2024-01

**Authors:** Elena Doering, Georgios Antonopoulos, Merle Hoenig, Thilo van Eimeren, Marcel Daamen, Henning Boecker, Frank Jessen, Emrah Düzel, Simon Eickhoff, Kaustubh Patil, Alexander Drzezga, for the Alzheimer’s Disease Neuroimaging Initiative and the DELCODE Study Group

**Affiliations:** 1 Department of Nuclear Medicine, Faculty of Medicine and University Hospital Cologne, University of Cologne, Cologne, Germany;; 2 German Center for Neurodegenerative Diseases, Bonn, Germany;; 3 Brain and Behavior, Research Center Juelich, Juelich, Germany;; 4 Institute of Systems Neuroscience, Heinrich Heine University, Duesseldorf, Germany;; 5 Molecular Organization of the Brain, Research Center Juelich, Juelich, Germany;; 6 Department of Psychiatry, Faculty of Medicine and University Hospital Cologne, University of Cologne, Cologne, Germany; and; 7 German Center for Neurodegenerative Diseases, Magdeburg, Germany

**Keywords:** machine learning, cognitive impairment, neuroimaging

## Abstract

Deviations of brain age from chronologic age, known as the brain age gap (BAG), have been linked to neurodegenerative diseases such as Alzheimer disease (AD). Here, we compare the associations of MRI-derived (atrophy) or ^18^F-FDG PET–derived (brain metabolism) BAG with cognitive performance, neuropathologic burden, and disease progression in cognitively normal individuals (CNs) and individuals with subjective cognitive decline (SCD) or mild cognitive impairment (MCI). **Methods:** Machine learning pipelines were trained to estimate brain age from 185 matched T1-weighted MRI or ^18^F-FDG PET scans of CN from the Alzheimer’s Disease Neuroimaging Initiative and validated in external test sets from the Open Access of Imaging and German Center for Neurodegenerative Diseases–Longitudinal Cognitive Impairment and Dementia studies. BAG was correlated with measures of cognitive performance and AD neuropathology in CNs, SCD subjects, and MCI subjects. Finally, BAG was compared between cognitively stable and declining individuals and subsequently used to predict disease progression. **Results:** MRI (mean absolute error, 2.49 y) and ^18^F-FDG PET (mean absolute error, 2.60 y) both estimated chronologic age well. At the SCD stage, MRI-based BAG correlated significantly with beta-amyloid_1-42_ (Aβ_1-42_) in cerebrospinal fluid, whereas ^18^F-FDG PET BAG correlated with memory performance. At the MCI stage, both BAGs were associated with memory and executive function performance and cerebrospinal fluid Aβ_1-42_, but only MRI-derived BAG correlated with phosphorylated-tau_181_/Aβ_1-42_. Lastly, MRI-estimated BAG predicted MCI-to-AD progression better than ^18^F-FDG PET–estimated BAG (areas under the curve, 0.73 and 0.60, respectively). **Conclusion:** Age was reliably estimated from MRI or ^18^F-FDG PET. MRI BAG reflected cognitive and pathologic markers of AD in SCD and MCI, whereas ^18^F-FDG PET BAG was sensitive mainly to early cognitive impairment, possibly constituting an independent biomarker of brain age-related changes.

## Visual Abstract

**Figure:**
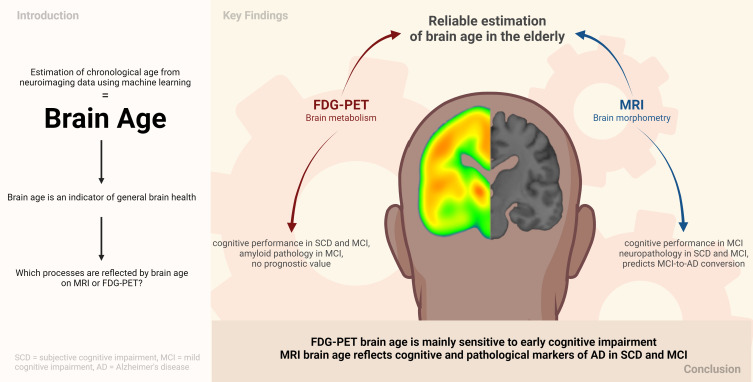

Brain aging entails changes in cognitive performance, brain function, and structural parameters of brain integrity. Brain age can be modeled using machine learning algorithms by estimating a person’s chronologic age from their neuroimaging data. Higher brain age than chronologic age, that is, a positive brain age gap (BAG), is associated with neurodegenerative diseases such as Alzheimer disease (AD). A recent study (1) linked BAG with PET AD biomarkers in patients with mild cognitive impairment (MCI) and with progression from cognitively normal (CN) to MCI, or MCI to dementia. This warrants further research on BAG as a marker of individual cognitive performance and neuropathologic burden in at-risk populations for AD dementia (subjective cognitive decline [SCD] and MCI).

Age-related changes are evident in the brain’s anatomy, such as loss of brain volume (atrophy), as well as in metabolism (neuronal dysfunction), which can be quantified by T1-weighted MRI and ^18^F-FDG PET, respectively. ^18^F-FDG PET is generally acknowledged as an earlier indicator of neurodegeneration than is structural MRI, as neuronal dysfunction precedes atrophy (i.e., neuronal loss). Moreover, regional proneness to age-related decline is different when assessed with ^18^F-FDG PET or MRI (2). Consequently, it is plausible to assume that an ^18^F-FDG PET–derived BAG is more sensitive to neuronal changes preceding neurodegeneration, such as neuropathologic burden or cognitive deficits below the threshold of AD. To date, however, brain age estimation (BAE) frameworks are almost exclusively modeled from MRI data. One recent study compared the 2 modalities and showed highly accurate BAE when using either MRI or ^18^F-FDG PET (1). This argues for further exploration of ^18^F-FDG PET–derived BAG and its performance in delineating the earliest deviations from normal aging in the absence of dementia.

Here, we investigated ^18^F-FDG PET– and MRI-derived BAE, with a particular focus on how BAG is associated with cognitive performance, neuropathologic burden, and disease progression in cognitively unimpaired individuals and MCI patients. First, we estimated brain age in cohorts of individuals who were CN, had subjective but not objective cognitive impairment (SCD), or showed MCI. Second, we calculated BAG and compared associations of ^18^F-FDG PET– or MRI-derived BAG with cognitive performance and AD neuropathology in these cohorts. Finally, we evaluated the prognostic value of BAG in predicting disease progression as compared with other established risk factors of cognitive decline.

## MATERIALS AND METHODS

The code used for this project is available on GitHub.

### Participants

Baseline T1-weighted MRI and ^18^F-FDG PET scans of 185 CNs (interscan interval, 28 ± 23 d) were acquired from the Alzheimer’s Disease Neuroimaging Initiative (ADNI) database (https://adni.loni.usc.edu/). For external validation, 49 MRI and ^18^F-FDG PET scans of CNs were acquired from the Open Access of Imaging Studies database, release 3 (3) (OASIS, https://www.oasis-brains.org/). We also assessed brain age in clinical samples of SCD (*n* = 102) and MCI (*n* = 595) patient groups from ADNI. The significant findings from these analyses were subsequently validated in SCD (^18^F-FDG PET, *n* = 88) and MCI (MRI, *n* = 80) samples from the German Center for Neurodegenerative Diseases–Longitudinal Cognitive Impairment and Dementia study (DELCODE) (4). CN, SCD, and MCI diagnoses from ADNI, OASIS, and DELCODE followed current recommendations for the respective groups (details are provided in Supplemental Section 1a; supplemental materials are available at http://jnm.snmjournals.org) (5*,*^6^). All participants gave written consent. Data collection was approved by local institutional review boards, and ethics proposals for retrospective dataset analysis were approved by Heinrich Heine University Düsseldorf.

### Estimation of Brain Age

**FIGURE 1.**
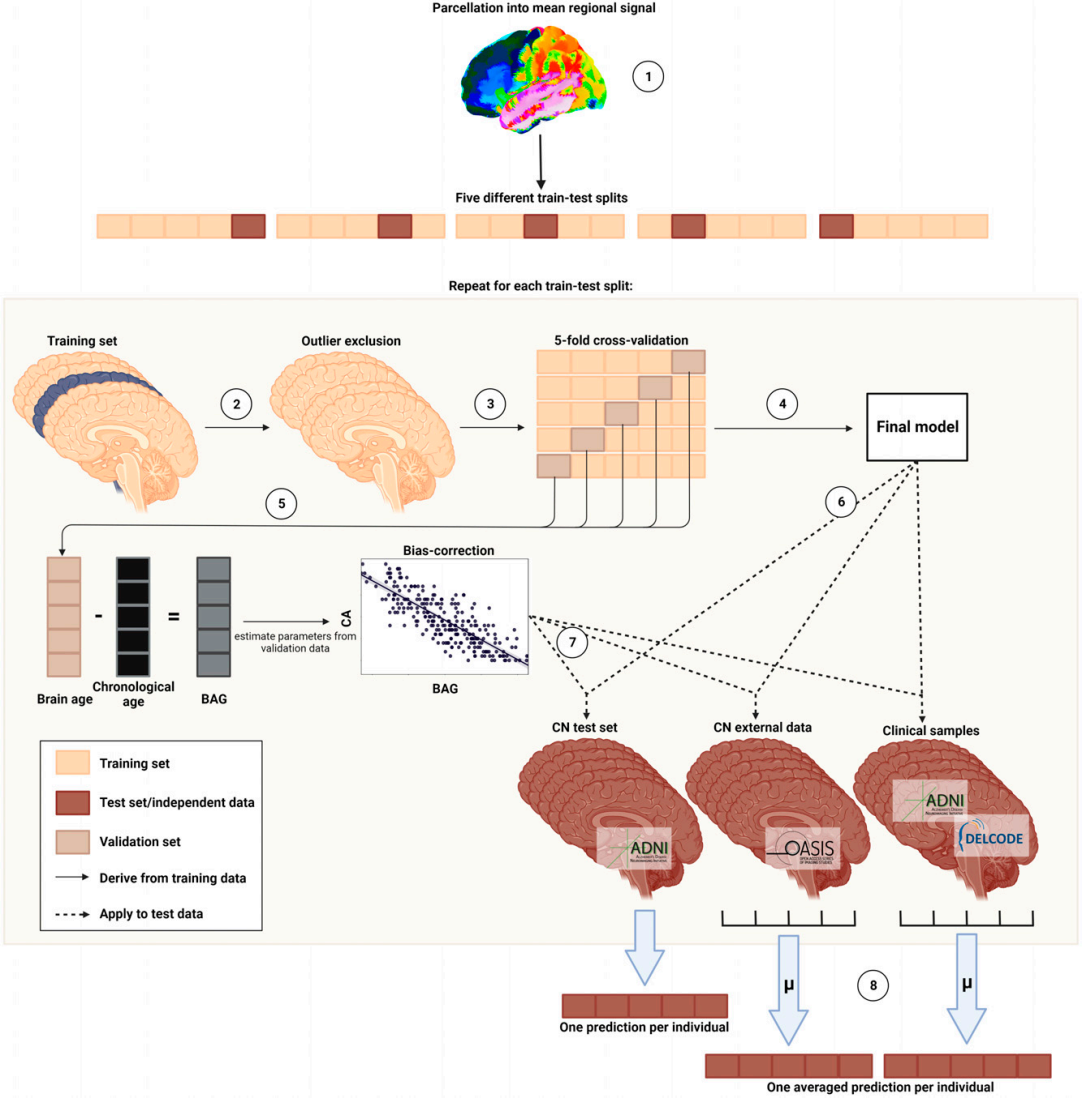
Nested cross-validation approach for brain age prediction. (1) Region-of-interest parcellation. (2) Outlier exclusion. (3) Five-fold cross-validation. (4) Final model selection. (5) Bias correction. (6) Estimation of brain age in test sets. (7) Bias correction in test sets. (8) Ensemble averaging. (Created with BioRender.com.)

Standardized MRI and ^18^F-FDG PET scans were used to compute brain age (details on acquisition and preprocessing are in Supplemental Section 1b). We implemented a pipeline (Fig. 1) in Python 3.8.5 using the Julearn library (https://juaml.github.io/julearn/main/index.html), which is based on scikit-learn (7). The same pipeline was run independently for MRI and ^18^F-FDG PET. First, a modality-specific signal of 90 cortical and subcortical regions of interest was extracted (MRI: gray matter volume; ^18^F-FDG PET: SUV ratio) using the automated anatomic labeling atlas (8). The atlas dependence of our results was assessed by repeating our analyses with a second composite atlas (Schaeffer + Tian atlas). We applied a nested cross-validation approach, with 5 folds in both the outer and the inner cross-validation. Outlier exclusion was performed in the outer cross-validation. Subsequently, support or relevance vector regression models, recommended for small sample sizes (9), were trained with hyperparameter optimization to compute brain age in the inner cross-validation loop. Selection of the final model across support and relevance vector regression models was based on the mean absolute error of the validation folds. Estimation of bias correction parameters was then based on predictions from the validation folds (10). The final model was used to estimate brain age in the test and clinical samples, and bias correction was applied (Supplemental Sections 1c–1e).

The nested cross-validation approach yielded one brain age per nonoutlier subject in the ADNI CNs, who were evenly spread across 5 test sets. Each cross-validation fold additionally yielded one estimate per subject in the OASIS and clinical samples; thus, the average of 5 estimates was treated as the final brain age (ensemble averaging).

### Statistical Analyses

BAG was calculated for each individual as the difference between brain age and chronologic age, such that higher BAG reflected more advanced brain age and vice versa.

#### Accuracy, Generalizability, and Variation of BAG

The accuracy of age estimation from MRI or ^18^F-FDG PET was assessed by comparing the mean absolute error of BAE across modalities using a paired t-test in the ADNI CN sample. To assess the generalizability of our BAE frameworks, we compared the mean absolute error of MRI- or ^18^F-FDG PET–based BAE between ADNI CNs and OASIS CNs using t-tests. Whether BAG was higher in the clinical populations was assessed by t-test comparisons of average BAG between ADNI CNs and each clinical sample.

#### Regional Importance

To understand the similarity of brain age models and to test whether AD-typical regions are relevant in BAE from MRI or ^18^F-FDG PET, we assessed Pearson correlations of BAG and feature importance (δ) across modalities in ADNI CNs. Feature importance was computed using permutation importance, with higher values corresponding to greater relevance of a feature for the model. For simplicity, we computed correlations using the average feature importance over all final models per modality. We further summarized regional feature importance per modality into median signal for lobes (frontal, temporal, limbic, subcortical, occipital, parietal; details are in Supplemental Section 1f), hemispheres (left, right), and lobes by hemisphere to assess whether brain regions of a particular category were preferred for BAE in a given modality.

#### Cognitive and AD-Neuropathologic Associations

To assess whether BAG is associated with cognitive performance, we calculated partial correlations between BAG and composite scores of memory (ADNI memory) (11) and executive function (ADNI executive function) (12) for the ADNI CN, ADNI SCD, and ADNI MCI groups (Supplemental Section 1g). In addition, partial correlations of BAG with PET amyloid load (^18^F-AV-45 PET) (13), and cerebrospinal fluid (CSF) markers (14) of beta-amyloid_1-42_ (Aβ_1-42_), and phosphorylated-tau_181_ (p-tau_181_)–to–Aβ_1-42_ ratio (p-tau_181_/Aβ_1-42_) (Supplemental Section 1h) (15) were calculated to assess whether BAG is associated with AD neuropathology. Pearson or Spearman correlations were assessed, depending on normality (Shapiro–Wilk test), and all partial correlations were corrected for age, sex, education, and APOE-ε4 carriership. Individuals with missing data for the dependent variable were excluded for each respective correlation. A *P* value of less than 0.1 was considered trend-significant, and a *P* value of less than 0.05 was considered significant. We also assessed the significance after Bonferroni correction (cognitive performance: α = 0.05/2, AD neuropathology: α = 0.05/3).

#### Disease Progression

Finally, we assessed whether BAG is associated with or even predicts disease progression. Relative to baseline BAG assessment, we differentiated between cognitively stable individuals, who maintained their baseline diagnosis until the 2-y follow-up, and decliners, who received a diagnosis of more severe cognitive impairment within follow-up (Supplemental Section 1i). Using analysis of covariance, the BAG between stable individuals and decliners was compared, while correcting for sex, education, and APOE-ε4 carriership in ADNI CNs and ADNI SCD subjects and additionally for age in ADNI MCI subjects (where bias correction did not eliminate the correlation between age and MRI BAG, Supplemental Section 2a). Subsequently, we trained multiple single-feature logistic regression classifiers using stratified 10-fold cross-validation to predict progression to AD in ADNI MCI from ^18^F-FDG PET BAG; MRI BAG; hippocampal volume (16); global ^18^F-AV-45 PET SUV ratio; ^18^F-FDG PET SUV ratio in the precuneus (17); ^18^F-FDG PET SUV ratio in a meta–region of interest, previously suggested relevant for the progression of AD (18); p-tau_181_/Aβ_1-42_ ratio; ADNI memory score; or age. Notably, the small number of decliners prevented the development of reliable predictive machine learning models in the ADNI CN and ADNI SCD groups. To correct for the effects of age, sex, education, and APOE status, standardized residuals were computed for all individuals and for each predictor variable using a linear model trained on the stable individuals in each training fold (19). We compared the mean area under the curve (AUC) obtained from the validation folds across all predictors. If the BAG of one modality predicted disease progression (AUC > 0.7), we derived a cutoff given the a priori probability of disease progression in each training fold and validated this cutoff in the DELCODE MCI cohort.

## RESULTS

### Participants

An overview of participant characteristics is shown in Table 1. OASIS CN, ADNI SCD, and DELCODE SCD subjects were significantly younger than the main ADNI CN cohort. The SCD and MCI cohorts further differed from ADNI CNs in terms of cognitive performance (ADNI MCI and DELCODE MCI), years of education (ADNI SCD and DELCODE MCI), amyloid status (DELCODE SCD and ADNI MCI), and APOE-ε4 carriership distribution (ADNI MCI and DELCODE MCI).

**Table 1.** Overview of Samples

Parameter	ADNI CN	OASIS CN	ADNI SCD	ADNI MCI	DELCODE SCD	DELCODE MCI
*n* total	186	49	102	595	88	80
Age at PET scan (y)	73.8 (6.46)	70.6 (5.07)*	72.3 (5.60)*	73.2 (6.93)	70.9 (5.57)*	NA
Age at MRI scan (y)	73.8 (6.44)	69.2 (4.98)*	72.3 (5.60)*	73.2 (6.92)	NA	73.4 (5.87)
Sex (female, %)	53% (0)	53% (0)	59% (0)	42% (2)*	36% (0)*	36% (0)*
MMSE score	29 (1.26)	29 (0.78)	29 (1.20)	28 (1.75)*	29 (1.03)	28 (1.67)*
Education (y)	16 (2.54)	16 (2.51)	17 (2.50)*	16 (2.67)	16 (3.00)	14 (3.06)*
CSF Aβ_1-42_–positive (%)	41% (27)	NA	35% (9)	64% (126)*	22% (28)*	38% (38)
APOE-ε4 carrier (%)	29% (1)	NA	31% (0)	49% (4)*	38% (3)	49% (0)*
Progression status decliner (%)	10% (32)	NA	12% (19)	25% (135)	NA	38% (12)

* Significantly different from ADNI CN. *P* < 0.05.

NA = not applicable.

Categorical data are percentages with number of individuals with missing information in parentheses; continuous data are means with SD in parentheses. Percentage of CSF Aβ_1-42_ status indicates percentage of amyloid-positive individuals according to established thresholds.

### Accuracy, Generalizability, and Variation of BAG

MRI and ^18^F-FDG PET estimated age with comparably high accuracy in ADNI CNs (mean absolute error, 2.49 for MRI and 2.60 for ^18^F-FDG PET; Table 2). Within-modality comparison of mean absolute error in OASIS CNs and ADNI CNs yielded no significant differences (2.92 for MRI OASIS and 2.54 for ^18^F-FDG PET OASIS), suggesting that our frameworks have high generalization performance to external datasets comprising CN populations. Average ^18^F-FDG PET–derived, but not MRI-derived, BAG was trend-significantly advanced in ADNI SCD subjects. Comparably, ^18^F-FDG PET BAG was significantly advanced in DELCODE SCD subjects. In all MCI samples, BAG was significantly higher than in ADNI CNs across modalities. Bias correction successfully eliminated the correlation of BAG and age with the exception of MRI-derived BAG in ADNI MCI individuals (Supplemental Table 3). Results using the composite atlas were largely comparable to those obtained here (Supplemental Table 4).

**TABLE 2.** Accuracy of Estimating Age from ^18^F-FDG PET and MRI Scans

Parameter	Modality	*n*	MAE	Range	BAG	*R* ^2^	Accuracy (MAE MRI vs. ^18^F-FDG PET)	Generalizability (MAE current vs. ADNI CN)	Brain age advancement (BAG current vs. ADNI CN)
ADNI CN	MRI	175*	2.49	−9.4 to 8.7	0.06	0.74	*t* = 0.48 (−0.33 to 0.55)	NA	NA
	^18^F-FDG PET	175*	2.60	−10.1 to 9.6	−0.10	0.70		NA	
OASIS CN	MRI	49*	2.92	−7.1 to 8.4	0.13	0.42	* t* = −0.94 (−1.20 to 0.43)	*t* = 1.16 (−0.31, 1.18)	*t* = 0.12 (−1.12, 1.26)
	^18^F-FDG PET	49*	2.54	−5.0 to 6.8	0.89	0.63		*t* = −0.18 (−0.64, 0.53)	*t* = 2.00^†^ (0.01, 1.97)
ADNI SCD	MRI	102	2.50	−6.6 to 7.0	0.11	0.69	*t* = 0.26 (−0.42 to 0.54)	NA	*t* = 0.11 (−0.73, 0.82)
	^18^F-FDG PET	102	2.56	−5.6 to 9.8	0.64	0.69		NA	*t* = 1.86^‡^ (−0.05, 1.53)
ADNI MCI	MRI	595	3.30	−10.5 to 13.5	2.16	0.65	*t* = −5.72^¶^ (0.95 to 0.46)	NA	*t* = 7.47^¶^ (1.55, 2.65)
	^18^F-FDG PET	595	2.59	−10.0 to 11.0	0.55	0.78		NA	*t* = 2.23^†^ (0.08, 1.22)
DELCODE SCD	^18^F-FDG PET	88	3.16	−2.7 to 9.3	2.77	0.52	NA	NA	*t* = 7.45^¶^ (2.11, 3.63)
DELCODE MCI	MRI	80	3.69	−5.1 to 11.6	2.89	0.38	NA	NA	*t* = 6.04^¶^ (1.90, 3.75)

* After outlier exclusion using CN train set (IQR > 6).

† *P* < 0.05.

‡ *P* < 0.1.

¶ *P* < Bonferroni correction.

MAE = mean absolute error.

Data in parentheses are 95% CIs.

### Regional Importance

**FIGURE 2.**
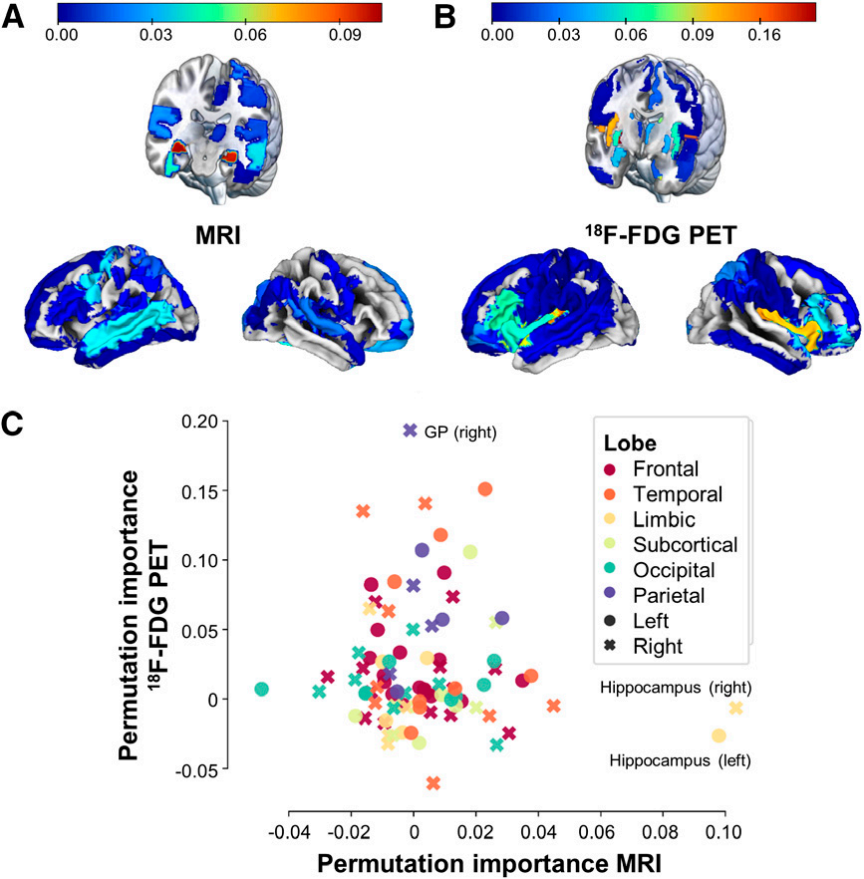
Feature importance for brain age prediction. (A and B) Average regional importance for brain age prediction using MRI (A) and ^18^F-FDG PET (B, threshold applied at 0). (C) Average feature importance across final models from ^18^F-FDG PET and MRI by lobe (colors) and hemisphere (shapes). GP = globus pallidus.

BAG was trend-significantly correlated between MRI- and ^18^F-FDG PET–based models (*r* = 0.128; *P* = 0.09; 95% CI, −0.02 to 0.27). Model selection returned different model types with mostly linear kernels (Supplemental Table 5). The left and right hippocampi were most relevant for MRI-based BAE (δ = 0.098 on left and 0.103 on right), whereas median permutation importance in the lobes, hemispheres, or lobes by hemisphere showed no obvious trends (Fig. 2). The subcortical regions (δ = 0.058: 0.058 on left and 0.067 on right) and, to a lesser extent, the left-hemispheric frontal (δ = 0.013) and temporal (δ = 0.012) regions were most relevant for ^18^F-FDG PET–based BAE. No overall hemispheric preference was observed for ^18^F-FDG PET models. Average regional importance did not correlate between MRI- and ^18^F-FDG PET–based models (*r* = −0.069; *P* = 0.52; 95% CI, −0.27 to 0.14).

### BAG and Cognitive Performance

**FIGURE 3.**
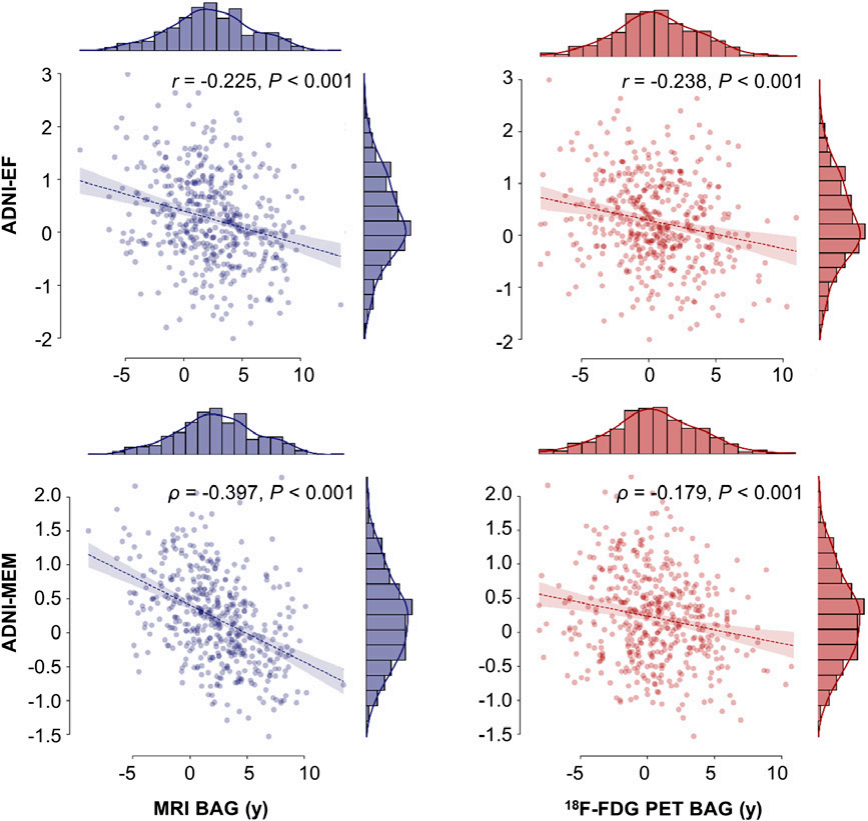
Correlation of BAG with cognitive performance in ADNI MCI. EF = executive function; MEM = memory. Correlations include correction for age, sex, and education.

In ADNI CNs, neither MRI nor ^18^F-FDG PET BAG was associated with cognitive performance (Table 3). In the ADNI SCD group, ^18^F-FDG PET BAG was significantly negatively associated with memory performance after Bonferroni correction and was trend-significantly associated with executive function. MRI BAG did not correlate with these measures (Table 3). In the ADNI MCI group, both MRI- and ^18^F-FDG PET–derived BAG was significantly negatively correlated with executive and memory performance after Bonferroni correction (Table 3; Fig. 3).

**TABLE 3.** Associations of BAG with Cognitive Performance and AD Neuropathology

		Cognitive performance		AD neuropathology		
Parameter	Modality	ADNI executive function	ADNI memory	^18^F-AV-45	CSF Aβ_1-42_	p-tau_181_/Aβ_1-42_
ADNI CN	MRI	*r* = 0.016 (−0.14 to 0.18)	*r* = −0.001 (−0.16 to 0.16)	ρ = −0.003 (−0.17 to 0.16)	ρ = 0.004 (−0.17 to 0.18)	ρ = 0.029 (−0.15 to 0.20)
	^18^F-FDG PET	*r* = 0.101 (−0.06 to 0.26)	*r* = 0.095 (−0.07 to 0.25)	ρ = 0.011 (−0.15 to 0.17)	ρ = −0.110 (−0.28 to 0.06)	ρ = 0.141 (−0.03 to 0.31)
ADNI SCD	MRI	*r* = 0.048 (−0.18 to 0.27)	*r* = −0.132 (−0.34 to 0.09)	ρ = 0.014 (−0.21 to 0.24)	*r* = −0.238* (−0.44 to −0.01)	ρ = 0.017 (−0.21 to 0.25)
	^18^F-FDG PET	*r* = −0.190^†^ (−0.39 to 0.03)	*r* = −0.259^‡^ (−0.45 to −0.04)	ρ = 0.191^†^ (−0.03 to 0.40)	*r* = −0.161 (−0.38 to 0.07)	ρ = 0.087 (−0.15 to 0.31)
ADNI MCI	MRI	*r* = −0.225^‡^ (−0.31 to −0.14)	ρ = −0.397^‡^ (−0.47 to −0.32)	ρ = 0.095^†^ (−0.01 to 0.02)	ρ = −0.230^‡^ (−0.32 to −0.13)	ρ = 0.200^‡^ (0.10 to 0.30)
	^18^F-FDG PET	*r* = −0.238^‡^ (−0.32 to −0.15)	ρ = −0.179^‡^ (−0.27 to −0.09)	ρ = 0.056 (−0.05 to 0.16)	ρ = −0.126* (−0.22 to −0.02)	ρ = 0.101^†^ (−0.00 to 0.20)

* *P* < 0.05.

† *P* < 0.1.

‡ *P* < Bonferroni correction.

*n* is described in Supplemental Tables 1 and 2. Data in parentheses are 95% CIs.

### BAG and AD Neuropathology

**FIGURE 4.**
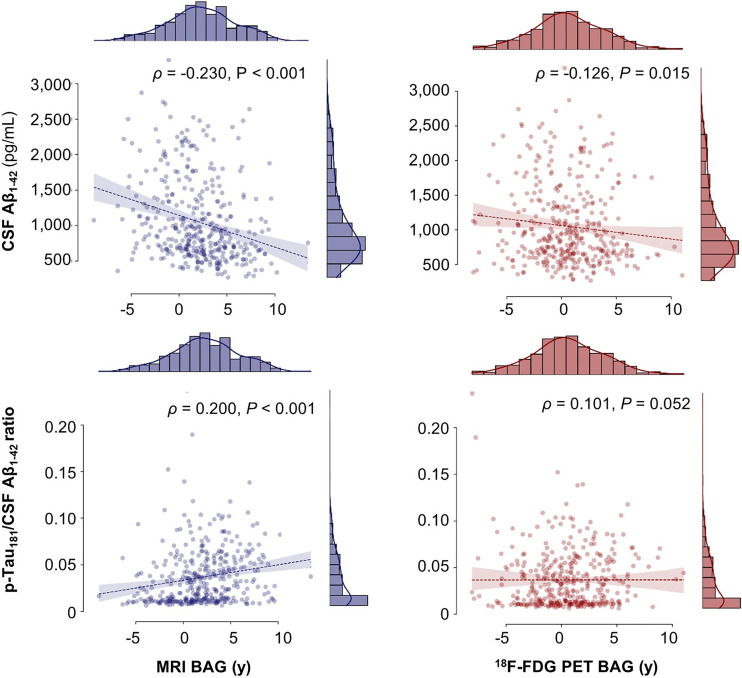
Correlation of BAG with AD neuropathology in ADNI MCI. Correlations include correction for age, sex, and education.

BAG and AD neuropathology did not significantly correlate in ADNI CNs. In the ADNI SCD group, lower levels of amyloid in CSF significantly correlated with increased MRI BAG, and a higher amyloid load in PET was trend-significantly associated with elevated ^18^F-FDG PET BAG (Table 3). In the ADNI MCI group, MRI BAG was trend-significantly correlated with all 3 markers of AD neuropathology, whereas ^18^F-FDG PET BAG was associated only with the CSF pathology markers (Table 3; Fig. 4).

### BAG and Disease Progression

**FIGURE 5.**
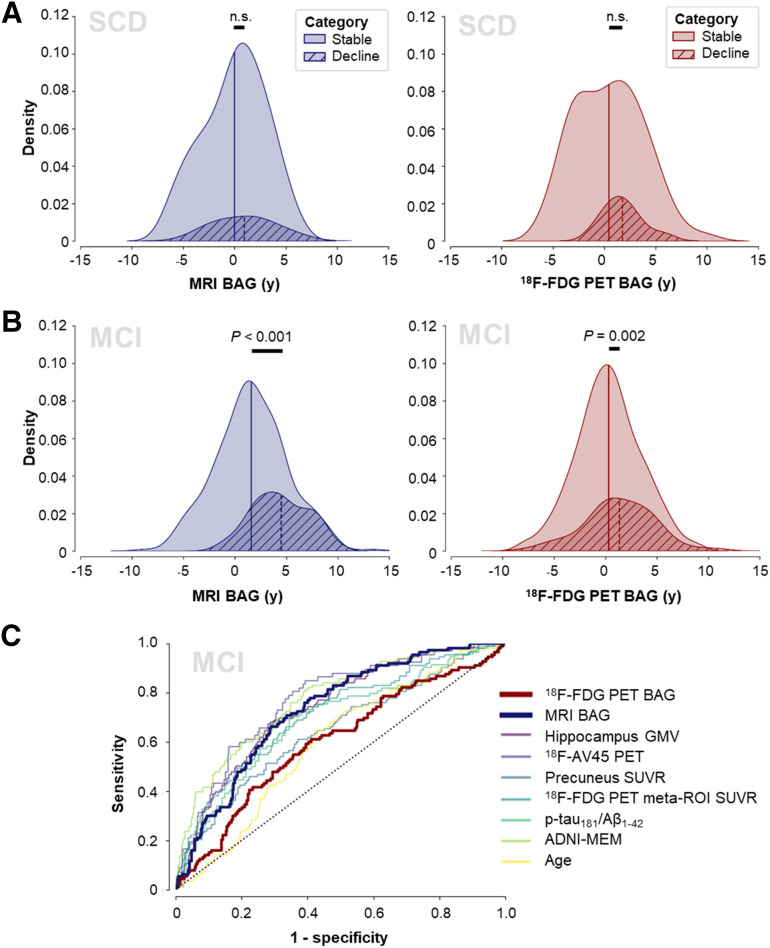
BAG for prediction of disease progression. Density plots show MRI and ^18^F-FDG PET BAG distribution by disease progression status in ADNI SCD (A) and ADNI MCI (B) and AUCs of prediction of disease progression (C). GMV = gray matter volume; MEM = memory; n.s. = not statistically significant; SUVR = SUV ratio.

Baseline BAG did not differ between stable individuals and decliners in the ADNI CN group (MRI BAG: *F*_1,149_ = 0.617, *P* = 0.43; ^18^F-FDG PET BAG: *F*_1,149_ = 0.023, *P* = 0.88; Supplemental Fig. 1) or the ADNI SCD group (MRI BAG: *F*_1,78_ = 0.247, *P* = 0.62; ^18^F-FDG PET BAG: *F*_1,78_ = 1.66, *P* = 0.20; Fig. 5A). In the ADNI MCI group, we found a significant main effect of group for both MRI and ^18^F-FDG PET BAG (MRI BAG: *F*_1,454_ = 59.64, *P* < 0.001; ^18^F-FDG PET BAG: *F*_1,454_ = 10.18, *P* = 0.002), with decliners showing advanced baseline BAG (MRI: mean of 4.51 y and SD of 2.79 y; ^18^F-FDG PET: mean of 1.35 y and SD of 3.38 y) compared with stable individuals (MRI: mean of 1.58 y and SD of 3.40 y; ^18^F-FDG PET: mean of 0.31 y and SD of 3.14 y; Fig. 5B). Men had a higher BAG than women across groups. Covariate effects are reported in detail in Supplemental Section 2d.

Next, we trained a logistic regression classifier to predict MCI-to-AD progression using corrected predictors. Progression to AD was predicted by MRI BAG (AUC, 0.73), ADNI memory (AUC, 0.78), ^18^F-AV-45 PET (AUC, 0.77), hippocampal volume (AUC, 0.75), SUV ratio in the ^18^F-FDG meta–region of interest (AUC, 0.72), and CSF p-tau_181_/Aβ_1-42_ ratio (AUC, 0.70). ^18^F-FDG PET BAG (AUC, 0.60) did not predict progression. Receiver operating characteristics are shown in Figure 5C. In the external DELCODE MCI cohort, MRI BAG predicted progression to AD with a similar AUC of 0.75. From a priori probabilities of cognitive decline in each training fold, we derived a mean probability cutoff of 0.25 in the MRI BAG–based model (range, 0.24–0.25), yielding sensitivities and specificities of 0.69 and 0.69, respectively, in the ADNI MCI group and 0.69 and 0.62, respectively, in the DELCODE MCI group.

## DISCUSSION

Previous studies mainly used MRI to estimate brain age. Here, we compared the accuracy of ^18^F-FDG PET– and MRI-estimated age and provided a comprehensive overview of the associations of BAG from either modality with cognitive performance, AD neuropathology, and disease progression in at-risk populations for AD dementia. Similar to Lee et al. (1), we found that BAE from the 2 modalities was comparably accurate. No associations were found for BAG and cognitive performance or neuropathologic burden in CNs. At the SCD stage, MRI-based BAG correlated significantly with CSF Aβ_1-42_, whereas ^18^F-FDG PET BAG, which was trend-significantly elevated in SCD, was linked to memory performance. In MCI, both BAGs were significantly elevated and associated with cognitive performance and CSF Aβ_1-42_. Only MRI-derived BAG correlated with p-tau_181_/Aβ_1-42_ and predicted MCI-to-AD progression.

MRI BAG was elevated in MCI patients compared with CNs and reflected AD neuropathologic burden both in SCD subjects and in MCI subjects. Moreover, MRI BAE was based mostly on hippocampal volume, a measure known to be associated with risk for dementia, and MRI BAG showed a moderate potential to predict MCI-to-AD progression. The increased association with CSF, compared with PET amyloid information, is likely due to the comparably early abnormality of CSF amyloid versus PET amyloid (20), as fluid amyloid biomarker signal disrupted amyloid metabolism whereas amyloid PET reveals resultant plaque aggregation. Therefore, MRI BAG closely reflects cognitive performance and relatively early pathologic markers of AD in SCD and MCI. The moderate predictive performance of not only MRI BAG but also the established biomarkers of MCI-to-AD progression suggests that a multimodal framework is required for an accurate prognosis in AD. To test the combined potential of established biomarkers and MRI BAG for risk assessment of AD, and possibly neurodegeneration in general, presents an interesting question for future research.

^18^F-FDG PET BAG was related to memory performance in SCD and MCI subjects but not in CNs. In SCD subjects, although memory performance is not yet objectively impaired, elevated ^18^F-FDG PET BAG may reflect its incipient decline. The regions we reported as relevant for ^18^F-FDG PET BAE are consistent with previous findings (1). Yet, we found no significant indications that ^18^F-FDG PET BAG is a prognostic biomarker. MRI and ^18^F-FDG PET BAG associations may differ because of regional differences in the BAE or the relative timing of MRI (atrophy) and ^18^F-FDG PET (neuronal dysfunction) changes in the course of AD. Future research is warranted to explore whether longitudinal ^18^F-FDG PET BAG may be valuable in tracking disease progression given its sensitivity to early cognitive impairment, its association with CSF amyloid burden in MCI, and the observed elevation of ^18^F-FDG PET BAG in SCD and MCI individuals.

Some limitations should be acknowledged. First, ^18^F-FDG PET BAG did not predict MCI-to-AD progression, although ^18^F-FDG PET itself is an established marker of AD progression (21). However, our algorithms were trained to estimate age, and the fact that relevant regions for ^18^F-FDG PET BAG did not include typical AD signature areas might explain this paradox. Second, although generalizability to OASIS data proved to be accurate, and despite training on multicentric data, we observed strong cohort effects for BAE in the external clinical samples. These results suggest that clinical and methodologic differences, such as variation in the extent of pathology, or in the diagnostic or scan procedure (e.g., the different acquisition times in DELCODE vs. ADNI or OASIS), can significantly influence the applicability of BAE frameworks. Finally, because of data availability and increased risk of cognitive deficits due to neurodegenerative processes, we included participants only over the age of 60 y. Thus, we did not investigate BAE before this age.

## CONCLUSION

BAE from MRI or ^18^F-FDG PET was highly accurate. MRI BAG reflected cognitive and pathologic markers of AD in SCD and MCI subjects, whereas ^18^F-FDG PET BAG related mainly to early cognitive impairment. Our study suggests that MRI BAG may especially complement the identification of patients who are likely to develop AD, whereas ^18^F-FDG PET BAG may represent a more independent biomarker of brain age-related changes, possibly occurring ahead of the clinical onset of neurodegeneration.

## DISCLOSURE

This work was partly supported by the Helmholtz Portfolio Theme “Supercomputing and Modelling for the Human Brain.” The DELCODE study was funded by the Deutsches Zentrum für Neurodegenerative Erkrankungen, German Center for Neurodegenerative Diseases (reference number BN012). Data collection and sharing for this project were funded by the ADNI (National Institutes of Health grant U01 AG024904) and the Department of Defense ADNI. Specific disclosures from ADNI and individual authors can be found in the supplemental materials. No other potential conflict of interest relevant to this article was reported.

## Notes

KEY POINTS **QUESTION:** How are BAGs that are derived from structural MRI or ^18^F-FDG PET linked to cognitive performance, AD neuropathology, and AD progression? **PERTINENT FINDINGS:** BAG was computed from structural MRI or ^18^F-FDG PET and subsequently associated with cognitive performance, neuropathologic markers of AD, and disease progression. ^18^F-FDG PET and MRI BAG were sensitive mainly to cognitive performance and amyloid burden in SCD, respectively. In MCI, both MRI and ^18^F-FDG PET BAG reflected cognitive performance and neuropathology. Finally, MRI BAG predicted MCI-to-AD conversion comparably well to established biomarkers. **IMPLICATIONS FOR PATIENT CARE:** Brain age is a thoroughly discussed concept, and its applicability must be evaluated in a modality- and group-specific manner. MRI-derived BAG may be particularly useful to complement the identification of patients who are likely to develop AD. MRI BAG may therefore benefit diagnostic procedures or clinical trials.
